# 

**Published:** 1908-12

**Authors:** 


					﻿Vol. XXIV.
DECEMBER, 1908.
No. 6.
TEXAS
JUDICAL
JOURNAL
“RED BACK”
PUBLISHED AT AUSTIN, TEXAS, B¥
F. E. DANIEL, M. D.,	-	- Proprietor
PRINTED BY THE VON BOECKMANN-JONES CO,
Subscription, $1.00 a Year, in Advance. -	-	- Single Copies, 10 Cents
Entered at the Postoffice at Austin, Texas, as Second-class Mail Matter
				

